# Supplementary material for: Survival of Campylobacter jejuni 11168H in Acanthamoebae castellanii Provides Mechanistic Insight into Host Pathogen Interactions
Source: Microorganisms. 2022 Sep 23;10(10):1894. doi: 10.3390/microorganisms10101894 (PMC9612045; doi:10.3390/microorganisms10101894)
Supplement: Supplementary file 1 [file microorganisms-10-01894-s001.zip › Supplementary_File_S1.pdf]

Supplementary File S1

Survival of *Campylobacter jejuni in Acanthamoebae castellanii* *provides mechanistic insight into host pathogen interactions*

Fauzy Nasher[1*]., Burhan Lehri[1]., Megan F Horney[1]., Richard Stabler[1]., Brendan W Wren[1*].

[1]Faculty of Infectious and Tropical Diseases, London School of Hygiene and Tropical Medicine, London, United Kingdom.

*Address correspondence to **Brendan W. Wren**, brendan.wren@lshtm.ac.uk

and **Fauzy Nasher**, fauzy.nasher1@lshtm.ac.uk

Figure S1:

a) b)

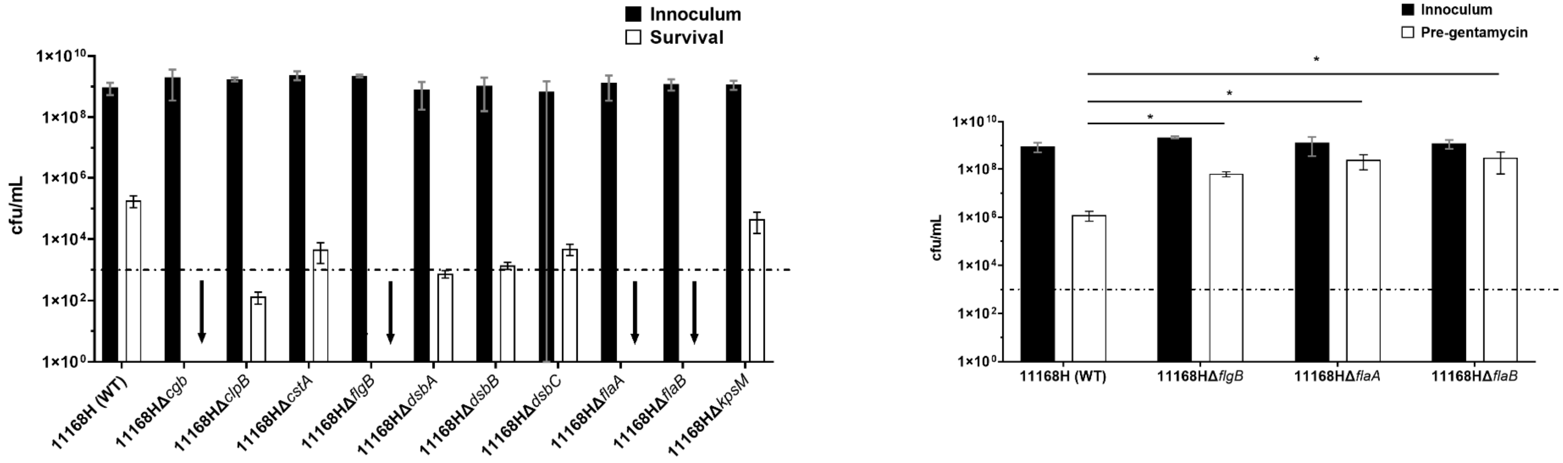

Figure S1: **Survival of *Campylobacter jejuni* 11168H and respective mutants within *Acanthamoeba castellanii*.** a) Amoebae were incubated with bacteria at an M.O.I of ~200:1 for 3 hrs before treatment with 100 µg/mL of gentamycin for 1 hr. Amoebae were lysed for enumeration of live bacteria. b) pre-gentamycin treatment enumeration to check for uptake of *11168H*Δcgb; *11168H*ΔflgB; *11168H*ΔflaA; and *11168H*ΔflaB by *A. castellanii*. Data is presented as cfu/mL; error bars represent SD from three independent experiments. Two-way ANOVA multiple comparison was used to test for significance; *$p \leq 0.05$.

Figure S2:

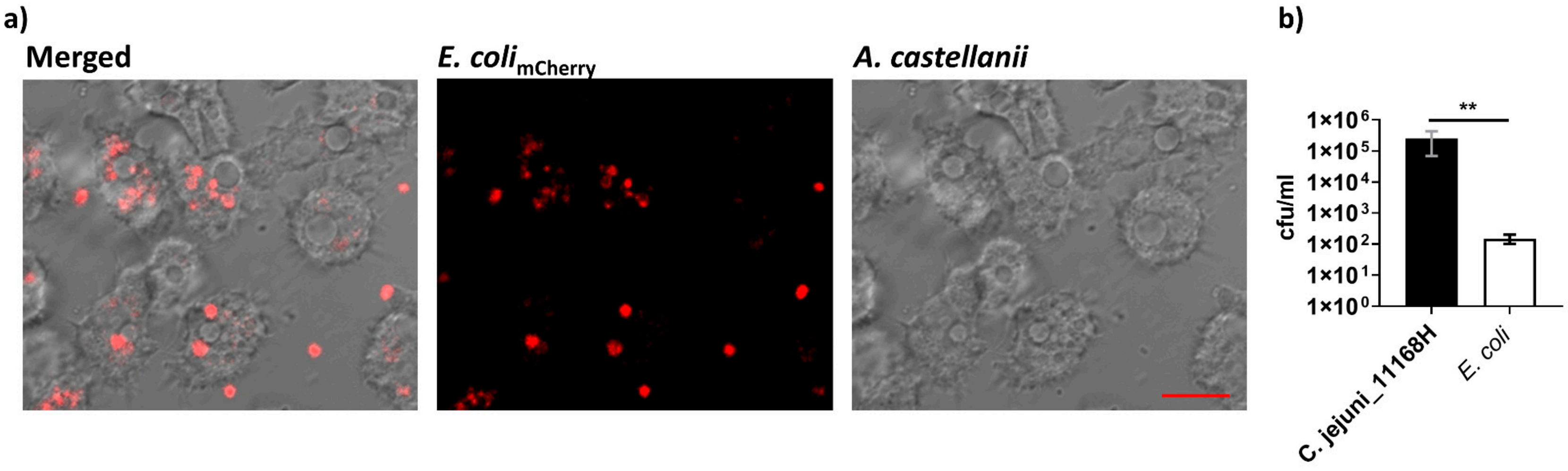

Figure S2: Survival of *E.*coli Dh5α$_{\text{mCherry}}$ within *A. castellanii*. a) Confocal microscopy images showing *E.coli*$_{\text{mcherry}}$ within *A. castellanii*; **b)** CFU of *E. coli*$_{\text{mCherry}}$ survival within *A. castellanii* after 4 hr infection compared to *C. jejuni* 11168H. Images represent merged transmitted light (amoebae); red channels (*E. coli*). Images were captured at x63 oil objective; scale bars: 10 µm; mCherry fluorescent was monitored in the red region (excitation and emission wavelengths are 587/610 nm). Error bars represent SD from three independent experiments; student *t*-test was used to test for significance **$p \leq 0.001$. Standard laboratory *E. coli* DH5α was transformed with a commercially available plasmid construct pFPV-mcherry, *E. coli* cells were acquired from New England Biolabs (USA) and transformation was performed according to manufactures protocol and selected with ampicillin.

Table S2: Table of all the strain used in this study.

**Strain**	**Antibiotic selection**
11168HΔ*cgb*	Kanamycin
11168HΔ*clpB*	Kanamycin
11168HΔ*cstA*	Kanamycin
11168HΔ*dsbA*	Kanamycin
11168HΔ*dsbB*	Kanamycin
11168HΔ*dsbC*	Kanamycin
11168HΔ*flaA*	Kanamycin
11168HΔ*flaB*	Kanamycin
11168HΔ*flgB*	Kanamycin
11168HΔ*kpsM*	Kanamycin
11168H$_{GFP}$	Chloramphenicol
11168H	N/A

Table S3: Primers used for RealTime RT-qPCR

Gene name	Primers for real-time RT-qPCR
***cgb* (*Cj1586*)**	F: 5'- GTTGCCATAACTCATGTTAATTTAGGAG-3'<br>R: 5'- CCAAGCTTTAAGAGTGGCTTCA-3'
***Ctb* (*Cj0465c*)**	F: 5' -CTATCATTTGTGCACGCTGTA-3'<br>R: 5' - AGCACTTAGATCTACCTCCTTT-3'
***ciaC* (*Cj1242*)**	F: 5'-GCAGATGAATTTCAAGCCACAT-3'<br>R: 5'- TTCTCTAACAGCACCAAGATCAA-3'
***Peb1A* (*Cj0921*)**	F: 5'-GCTATCACCGCATCTACACTAC-3'<br>R: 5'- GTTTCGAAGTAGATGTTGCCAAA-3'
Cj0971	F: 5'-CAGCTGATGAACTAACAAGCATTA-3'<br>R: 5'- CAATACTTCCTTTAAAGCGTTTGC-3'
***dsbA* (*Cj0872*)**	F: 5' -CTCTATCCTGTAAGTTTAATGAATGGG-3'<br>R: 5'- CTATCAGAATAACTCGCATCTTTACC-3'
**(*cstA*) (*Cj0917*)**	F: 5'-CCCAATAAGGATCAGCAGCTATAA-3'<br>R: 5'- TTATCCGTCCAGGTAGAGTAGG-3'
***clpB* (*Cj0509c*)**	F: 5'-CGGTTGCTCTACCATCATCTAA-3'<br>R: 5'- CTTACTGAAGCCGTACGAAGAA-3'
***mrdB* (*Cj1282*)**	F: 5'-AGCTATAAGTGTTAGCTCTCCTATT-3'<br>R: 5'- TTTCCGGTTAAACCACCATTTC-3'
***kpsM* (*Cj1448*)**	F: 5' -GCAAAGTTCTTAAAGGTTCCACA-3'<br>R: 5' - CCTGTTCATTTGCTTGGAGTTT-3'
***mrsA* (*Cj0637c*)**	F: 5'-CTTCTATGTTGCCATCACCATTAC-3'<br>R: 5' - AGCCGTATTTGAACGCCTAA-3'
Cj1276c	F: 5' -CCGTATTTACACACAACAAGCA-3'<br>R: 5' - CCAAGAAAGTTTAAAGGCTGTAGAT-3'
***gyrA* (*Cj1027c*)**	F: 5' -AGTAATACGTGGCACATCAAATTTACTTCTAAT-3'<br>R: 5'- GCAGAATTAATGAAAGAAATTGCAAGACTTG-3'
***ciaI* (*Cj1450*)**	F: 5' -GAAGGCTCTAAGCTCACACAA-3'<br>R: 5' - TGGCTTTAACTCTCCGACTTTAG-3'
***dsbB* (*Cj0865*)**	F: 5'-GCAAGATCTATTGTTGTATGTGCTT-3'<br>R: 5'- CACTATAATTATCAGCGTTAGCAATAGG-3'

F= Forward primer
R= Reverse primer
